# Rising Serum Uric Acid Level Is Negatively Associated with Survival in Renal Cell Carcinoma

**DOI:** 10.3390/cancers11040536

**Published:** 2019-04-15

**Authors:** Kendrick Yim, Ahmet Bindayi, Rana McKay, Reza Mehrazin, Omer A. Raheem, Charles Field, Aaron Bloch, Robert Wake, Stephen Ryan, Anthony Patterson, Ithaar H. Derweesh

**Affiliations:** 1 Department of Urology, University of California at San Diego, La Jolla, CA, 92093, USA; keyim@ucsd.edu (K.Y.); ahmetbindayi@gmail.com (A.B.); rmckay@ucsd.edu (R.M.); oraheem@uw.edu (O.A.R.); c1field@ucsd.edu (C.F.); abloch@ucsd.edu (A.B.); stryan@ucsd.edu (S.R.); 2 Department of Urology, University of Tennessee Health Sciences Center, Memphis, TN 38163, USA; Reza.mehrazin@mountsinai.org (R.M.); rwake@uthsc.edu (R.W.); apatterson@uthsc.edu (A.P.)

**Keywords:** chronic kidney disease, nephrectomy, overall survival, recurrence free survival, renal cell carcinoma, statins, uric acid

## Abstract

*Aim and Background:* To investigate the association of serum uric acid (SUA) levels along with statin use in Renal Cell Carcinoma (RCC), as statins may be associated with improved outcomes in RCC and SUA elevation is associated with increased risk of chronic kidney disease (CKD). *Methods:* Retrospective study of patients undergoing surgery for RCC with preoperative/postoperative SUA levels between 8/2005–8/2018. Analysis was carried out between patients with increased postoperative SUA vs. patients with decreased/stable postoperative SUA. Kaplan-Meier analysis (KMA) calculated overall survival (OS) and recurrence free survival (RFS). Multivariable analysis (MVA) was performed to identify factors associated with increased SUA levels and all-cause mortality. The prognostic significance of variables for OS and RFS was analyzed by cox regression analysis. *Results:* Decreased/stable SUA levels were noted in 675 (74.6%) and increased SUA levels were noted in 230 (25.4%). A higher proportion of patients with decreased/stable SUA levels took statins (27.9% vs. 18.3%, *p* = 0.0039). KMA demonstrated improved 5- and 10-year OS (89% vs. 47% and 65% vs. 9%, *p* < 0.001) and RFS (94% vs. 45% and 93% vs. 34%, *p* < 0.001), favoring patients with decreased/stable SUA levels. MVA revealed that statin use (Odds ratio (OR) 0.106, *p* < 0.001), dyslipidemia (OR 2.661, *p* = 0.004), stage III and IV disease compared to stage I (OR 1.887, *p* = 0.015 and 10.779, *p* < 0.001, respectively), and postoperative de novo CKD stage III (OR 5.952, *p* < 0.001) were predictors for increased postoperative SUA levels. MVA for all-cause mortality showed that increasing BMI (OR 1.085, *p* = 0.002), increasing ASA score (OR 1.578, *p* = 0.014), increased SUA levels (OR 4.698, *p* < 0.001), stage IV disease compared to stage I (OR 7.702, *p* < 0.001), radical nephrectomy (RN) compared to partial nephrectomy (PN) (OR 1.620, *p* = 0.019), and de novo CKD stage III (OR 7.068, *p* < 0.001) were significant factors. Cox proportional hazard analysis for OS revealed that increasing age (HR 1.017, *p* = 0.004), increasing BMI (Hazard Ratio (HR) 1.099, *p* < 0.001), increasing SUA (HR 4.708, *p* < 0.001), stage III and IV compared to stage I (HR 1.537, *p* = 0.013 and 3.299, *p* < 0.001), RN vs. PN (HR 1.497, *p* = 0.029), and de novo CKD stage III (HR 1.684, *p* < 0.001) were significant factors. Cox proportional hazard analysis for RFS demonstrated that increasing ASA score (HR 1.239, *p* < 0.001, increasing SUA (HR 9.782, *p* < 0.001), and stage II, III, and IV disease compared to stage I (HR 2.497, *p* < 0.001 and 3.195, *p* < 0.001 and 6.911, *p* < 0.001) were significant factors. *Conclusions*: Increasing SUA was associated with poorer outcomes. Decreased SUA levels were associated with statin intake and lower stage disease as well as lack of progression to CKD and anemia. Further investigation is requisite.

## 1. Introduction

There is increasing evidence that renal cell carcinoma (RCC) is a metabolically driven disease. Many of the known genes associated with the development of RCC are involved in regulating cellular metabolism within nutrient-deprived tumor microenvironments [1,2,3]. Moreover, recent studies have identified components of the metabolic syndrome (hypertension, hyperglycemia, hyper-triglyceridemia, and obesity) as independent risk factors for developing RCC [4,5,6]. These same metabolic derangements are also risk factors for developing chronic kidney disease (CKD) [7,8], with higher morbidity and mortality in CKD patients undergoing extirpative surgery for RCC [9].

Uric acid is the final breakdown product of purine metabolism and is associated with significant health problems. Hyperuricemia, defined as serum uric acid (SUA) elevation, is correlated with development of atherosclerosis, metabolic syndrome, and of CKD after surgery for renal tumors [10,11]. Consequently, SUA elevation may be exploited as a biomarker of disease risk at the intersection of these critical pathophysiologic processes. 

A growing body of literature suggests that receiving of statin (HMG-CoA reductase inhibitor) medications may be associated with improved outcomes in RCC through its purported anti-neoplastic activity and renoprotective properties [12,13]. Furthermore, recent reports suggest that SUA may be a marker of response to statin therapy [14]. Different types of statins have various efficacies at reducing SUA levels, and prolonged high-intensity statin therapy has been shown to preserve kidney function and has been associated with decreased SUA levels. [15,16]. In this study, we sought to investigate the relationship between receipt of statin medications, SUA levels, and outcomes in patients with RCC. We hypothesized that increasing uric acid was associated with adverse renal functional and metabolic endpoints which correlated with decreased survival, and that statin therapy was associated with a decreased risk of elevated SUA levels. 

## 2. Results

A total of 905 patients were identified with appropriate SUA data between August 2005 and August 2018. Table 1 lists demographic and clinical disease characteristics. 

610 patients underwent radical nephrectomy and 295 underwent partial nephrectomy. Decreased/stable SUA levels were noted in 675 (74.6%) and increased SUA levels were noted in 230 (25.4%). Patients with increased SUA levels were more likely to male (*p* = 0.0393) and obese (*p* = 0.0201). A total of 230 (25%) patients took statins medication. A significantly greater proportion of patients with decreased/stable SUA levels were taking statins (27.9% vs. 18%, *p* = 0.004), had localized RCC (Clinical Stage I/II disease, *p* < 0.011), or underwent nephron sparing surgery (*p* < 0.001). 

Table 2 summarizes the renal function and metabolic outcomes in the increased SUA and decreased/stable SUA groups. Patients with increased SUA were more likely to develop de novo eGFR < 60 (38.7% vs. 18.4%, *p* < 0.0001). In addition, patients with increased SUA were more likely to have postoperative proteinuria (30.9% vs. 20.7%, *p* = 0.0017), metabolic acidosis (20.9% vs. 11.7%, *p* = 0.0005), and anemia (47% vs. 25.3%, *p* < 0.0001) when compared to patients with decreased/stable SUA. 

In Table 3, UVA and MVA regression analysis were completed for factors associated with increased postoperative SUA. UVA showed that male sex, increasing BMI, statin utilization, dyslipidemia, increasing AJCC stage, RN, and de novo CKD stage III were all significantly associated with increased postoperative SUA. MVA revealed that statin utilization (OR 0.106, 95%CI 0.06–0.19, *p* < 0.001), increasing BMI (OR 1.05, 95% CI 1.01–1.09, *p* = 0.009), dyslipidemia (OR 2.66, 95% CI 1.36–5.2, *p* = 0.004), AJCC stage III and IV disease compared to stage I (OR 1.89, 95% CI 1.13–3.15, *p* = 0.015 and 10.78, 95% CI 4.07–28.52, *p* < 0.001, respectively), and postoperative de novo CKD stage III (OR 5.95, 95% CI 3.95–8.96, *p* < 0.001) were predictors for increased postoperative SUA levels.

In Table 4, UVA and MVA were also completed to identify risk factors for overall mortality. UVA showed that male gender, increasing BMI, increasing American society of Anesthesiologists’ physical status classification (ASA score), increased SUA, increasing AJCC stage, de novo CKD stage III, and RN were associated with overall mortality. On MVA, increasing BMI (OR 1.09, 95% CI 1.03–1.14, *p* = 0.02), increasing ASA Score (OR 1.57, 95% CI 1.10–2.27, *p* = 0.014), increased SUA (OR 4.70, 95% CI 2.94–7.50, *p* < 0.001), stage IV compared to stage I disease (OR 7.70, OR 2.87–20.63, *p* < 0.001), RN compared to PN (OR 1.62, 95% CI 1.08–2.42, *p* = 0.019), and de novo CKD stage III (OR 7.07, 95% CI 5.09–9.81, *p* < 0.001) were all significant predictors for all-cause mortality.

Table 5 demonstrates the result of a Cox regression analysis to investigate the association between survival time and a number of predictor variables. In the univariate analysis, increasing age, male sex, increasing BMI, increasing ASA score, increased SUA, stages II, III, and IV compared to stage I, RN compared to PN, and de novo CKD stage III were associated with survival time. On MVA, age (HR 1.02, 95% CI 1.005–1.028, *p* = 0.004), increasing BMI (HR 1.10, 95% CI 1.07–1.13, *p* < 0.001), increased SUA (HR 4.71, 95% CI 3.65–6.08, *p* < 0.001), stage III (HR1.54, 95% CI 1.10–2.16, *p* = 0.013) and IV (HR 3.29, 95% CI 1.92–5.67, *p* < 0.001) compared stage I disease, RN compared to PN (HR 1.50, 95% CI 1.04–2.15, *p* = 0.029), and de novo CKD stage III (HR 1.68, 95% CI 1.30–2.19, *p* < 0.001) were independent prognostic factors for survival time. 

Table 6 shows the result of a Cox regression analysis to investigate the association between time to recurrence and a number of predictor variables. In the univariate analysis, male sex, BMI, ASA score, increased SUA, stage II, III, and IV compared to stage I, RN compared to PN, and de novo CKD stage III were significantly associated with time to recurrence. On MVA, increased SUA (HR 9.78, 95% CI 6.48–14.77, *p* < 0.001), stage II (HR 2.49, 95% CI 1.58–3.94, *p* < 0.001), stage III (HR 3.19, 95% CI 1.99–5.13, *p* < 0.001) and IV (HR 6.91, 95% CI 3.95–12.08, *p* < 0.001) compared stage I disease were independent prognostic factors for recurrence free survival. In Table 7, we observed a positive correlation between clinical stage and increased SUA (*r* = 0.188, *p* < 0.001) and an inverse relationship between survival and increased SUA (*r* = −0.317, *p* < 0.001).

**Figure 1:**
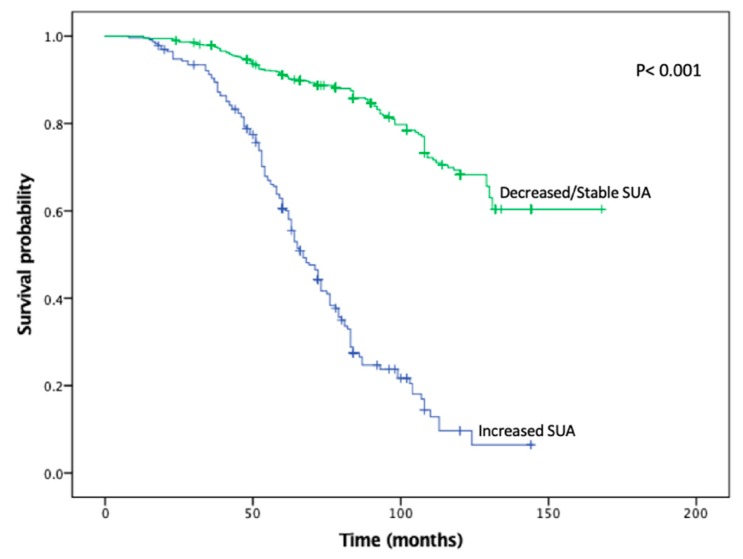
Kaplan-Meier plot for overall survival in the stable/decreased uric acid and increased uric acid groups.

**Figure 2:**
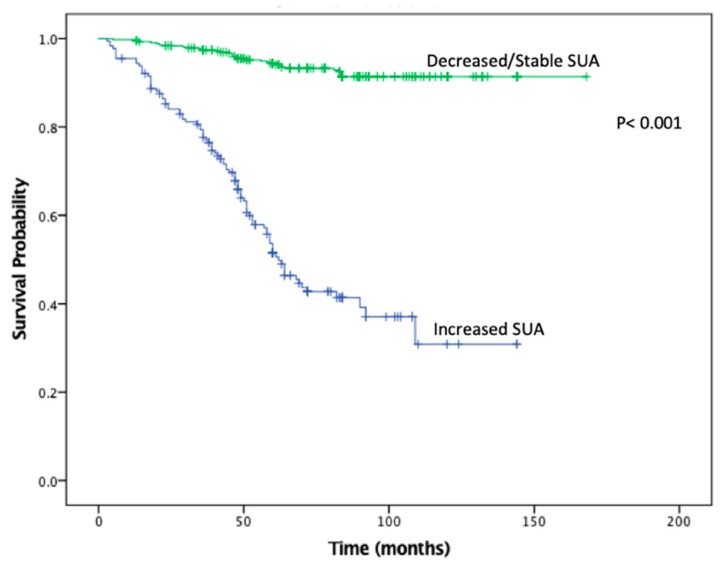
Kaplan-Meier plot for recurrence free survival in the stable/decreased uric acid and increased uric acid groups.

**Figure 3:**
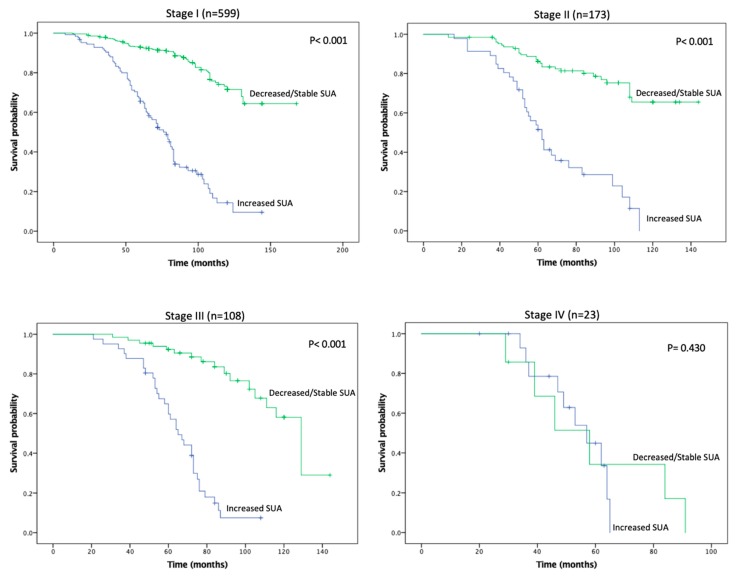
Kaplan-Meier plot for overall survival in the stable/decreased uric acid and increased uric acid groups, separated by disease stage.

**Figure 4:**
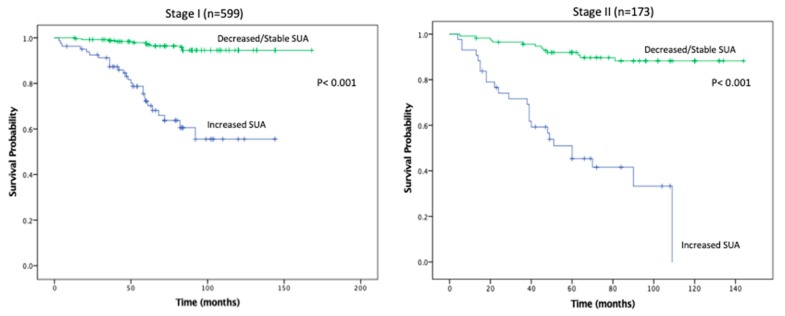

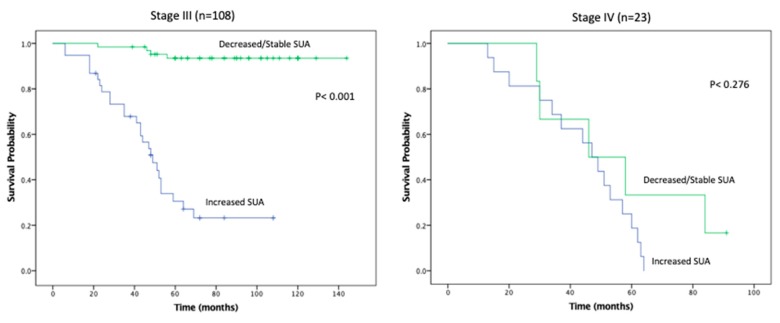
Kaplan-Meier plot for recurrence free survival in the stable/decreased uric acid and increased uric acid groups, separated by disease stage.

KMA demonstrated improved 5- and 10-year OS (89% vs. 47% and 65% vs. 9%, *p* < 0.001; Figure 1) and RFS (94% vs. 45% and 93% vs. 34%, *p* < 0.001; Figure 2), favoring patients with decreased/stable SUA levels. Estimated rates of OS at 5 years following surgery for the patients with elevated SUA with stage I, II and III disease were 55%, 37%, and 44%, respectively, compared with 91%, 83%, and 91%, respectively, for the patients with decreased/stable SUA (*p* < 0.001; Figure 3). There was no significant OS difference between patients with stage IV disease (*p* = 0.437; Figure 3). Estimated rates of RFS at 5 years following surgery for patients with elevated SUA with stage I, II, and III disease were 66%, 41%, 22% respectively, compared with 98%, 89%, 94%, (*p* < 0.001; Figure 4). There were no significant RFS difference between patients with stage IV disease (*p* = 0.276; Figure 4) 

## 3. Discussion

This current study represents the first large and multi-institutional analysis examining changes in preoperative and postoperative SUA as a prognostic marker of survival and renal function. Our findings suggest that changes in SUA levels can predict postoperative renal function, CKD sequelae, and survival in the context of RCC treated with nephrectomy. Patients with increased SUA had increased rates of de novo CKD, proteinuria, metabolic acidosis, osteoporosis, and anemia. In addition, postoperative increase in SUA was predictive of decreased OS and RFS in Stage I-III RCC. Lastly, patients taking statins were associated with decreased SUA and this cohort had significantly longer OS and RFS. 

Changes in SUA have been shown to correlate with cancer mortality in a number of non-urologic malignancies through both retrospective and prospective studies. In addition, various preclinical studies have demonstrated that increased intracellular SUA may induce the inflammatory stress response, while elevated extracellular SUA stimulates various transcription factors that promote cellular proliferation, survival, and migration. These cellular changes facilitate the transformation of normally quiescent cells to highly aggressive cancer cells [17]. A retrospective study by Yue et al. analyzed 443 primary breast cancer patients and found that elevated SUA was associated with inferior overall survival (HR 2.13, *p* = 0.016) and was an independent prognostic factor for predicting death [18]. Similarly, a meta-analysis examining 12 prospective studies spanning 632,472 subjects by Yan et al. found that high SUA levels were associated with increased risk of cancer mortality (RR = 1.19, *p* = 0.010), with a larger effect in females (RR= 1.25, *p* = 0.004) in a variety of different cancers [19]. Yuki et al. conducted a retrospective analysis of 89 patients with RCC and noted that an increase of greater than 10% in SUA was predictive for metastases (*p* < 0.0001) [20]. This was the first study to associate increased serum uric acid with oncological endpoints in renal cell carcinoma. We have confirmed these findings by noting that an increase of 10% of more of SUA was independently predictive for RFS (HR 9.782, *p* < 0.001). In addition, our KMA demonstrated improved OS and RFS in patients with decreased/stable SUA levels at both 5 and 10 years. Our subset survival analysis showed improved OS and RFS in the decreased/stable SUA group within stage I-III disease. Expanding upon the work of Yuki et al., we report the first large-scale, multi-institutional study that identifies postoperative SUA as a negative predictor for survival in patients undergoing PN or RN for RCC.

Recent epidemiological studies have demonstrated an independent association between elevated SUA and increased CKD, although the interaction between increased SUA and CKD in the setting of RCC is not well understood [21,22,23]. Jeon et al. reviewed data from 1534 patients and found that increased baseline SUA was associated with lower preoperative GFR in patients with RCC (*r* = −0.313, *p* < 0.001). In addition, hypertension (OR 1.37, *p* = 0.075) and elevated SUA (OR 1.23, *p* = 0.002) were associated with new onset CKD in patients who underwent PN or RN for RCC [24]. The authors concluded that preoperative SUA might be able to predict CKD after extirpative intervention in patients with RCC. Similarly, in a prospective observational study, Obermayr et al. found that in a cohort of 21,475 patients, those with SUA > 7 mg/dL were nearly twice as likely to develop CKD (OR 1.74, *p* < 0.05), while those with SUA > 9 mg/dL were associated with a tripled risk (OR 3.12, *p* < 0.05) [25]. Instead of looking at SUA as a continuous variable, we stratified postoperative SUA as either increased or decreased/stable in order to better account for the temporal changes in SUA associated with surgical intervention. Our findings are consistent with the findings of these previous reports which demonstrated that patients with postoperative increases in SUA had greater risk for development of de novo CKD. Furthering these findings, we demonstrated that patients with increased SUA had increased frequency of CKD-associated sequelae including: metabolic acidosis, osteoporosis, and anemia. Indeed, our findings and those of Jeon et al. and Overlay et al. suggest that association of rising SUA with CKD and its sequelae may point towards development of surveillance and preventive strategies to identify patients at risk for renal functional degeneration and to attempt to attenuate CKD sequelae by early intervention. 

Recent investigations have also focused on identifying interventions that decrease the postoperative drops in eGFR in an effort to reduce complications associated with nephron loss in RN and PN. In a prospective, randomized trial of 54 hyperuricemic patients, Kose et al. found that atorvastatin significantly increased eGFR from 51.1 to 61.8 mL/min/1.73 m^2^, while significantly decreasing SUA from 6.38 mg/dL to 5.48 mg/dL in CKD patients [26]. Siu et al. demonstrated that patients treated with 100 to 300 mg of allopurinol had significantly decreased SUA in patients with mild to moderate CKD (9.75 mg/dL to 5.88 mg/dL, *p* < 0.001) and had slower progression of CKD [27]. In addition, statins have been studied for their ability to decrease SUA in addition to other renoprotective effects. A meta-analysis of 88,523 participants by Geng et al. found that statins reduced the decrease in eGFR (SMD 0.14, *p* = 0.007) and decreased proteinuria (SMD −0.19, *p* = 0.005) in patients with early-stage CKD after 3 years of therapy [28]. Although the mechanism of action of statins on SUA has not yet been elucidated, stronger statins such as atorvastatin are more lipophilic thus are more potent at improving endothelial function and increasing glomerular filtration [17]. Additionally, it is thought that statins increase decrease proximal tubular reabsorption of uric acid and this increase urinary uric acid excretion. Our studies approached this question from a different perspective and in a slightly different patient population. We looked at the relationship between statin use and increased or decreased/stable SUA in patients that had undergone extirpative surgery for RCC. Consistent with previous studies, we found that patients taking statins were nearly 10× less likely to have an increased postoperative SUA. 

In addition to lowering SUA, improving eGFR and reducing CKD progression, statins may also improve survival in patients with RCC. Recently, Hamilton et al. evaluated the effect of statin medications on RCC progression in a cohort of 2608 patients with localized RCC treated over a 15-year period. Of these patients, 699 (27%) were statin users at surgery. With a median follow-up of 36 months, they noted that statin use attenuated the risk of tumor progression to 23% (hazard ratio 0.77; *p* = 0.12) and augmented the risk reduction in overall survival (hazard ratio 0.71; *p* = 0.002) [29]. In addition, a retrospective study by McKay et al. analyzed 4736 patients treated for metastatic RCC and found that statin use was associated with improved survival (25.6 vs. 18.9 months, *p* = 0.025) [30]. Similarly, in our study, a total of 230/905 (25%) patients received statins and a higher proportion of patients with decreased/stable SUA levels were on statins (28% vs. 18%, *p* = 0.004). Taken together, the findings of McKay et al. and Hamilton et al. suggest a salutory effect of statin agents on RCC outcomes. Furthermore, our findings which demonstrated that decreased/stable SUA correlated with improved OS and RFS, and that receipt of statins was inversely correlated with elevated SUA, suggest utility of SUA as a marker of response and/or efficacy of statin agents in risk reduction for RCC patients. 

The present study is limited by its retrospective design, which has inherent potential for selection biases. In addition, because this was not a prospective analysis, we were unable to examine the duration of statin therapy and its potential effects of SUA levels. Nonetheless, our study is strengthened by its multicenter design and validated by our multivariable analysis results. This represents the first attempt to utilize SUA as a biomarker for OS and RFS in RCC. In addition, we were able to confirm that statin therapy is associated with decreased SUA and can predict CKD progression. Ultimately, additional prospective investigation is required to validate these findings, further elucidate the molecular interaction between SUA and RCC and characterize SUA’s clinical utility. 

## 4. Materials and Methods 

### 4.1. Study Population

This study was conducted in accordance with the Declaration of Helsinki, and the protocol was approved by the Institutional Review Boards of The University of California (San Diego, CA, USA), and The University of Tennessee Health Science Centre (Memphis, TN, USA). Pre- and postoperative data of 905 patients with RCC who underwent surgery between 2005 and 2018. Patients with no prior preoperative or postoperative SUA levels, history of gout, receipt of allopurinol, or incomplete records were excluded. Six week preoperative and 6 week postoperative SUA levels were recorded. Demographics, clinical characteristics, renal function and oncological outcomes were analyzed and compared.

### 4.2. Study Design

The primary endpoint was overall survival (OS). Secondary endpoints were recurrence free survival (RFS), development of CKD (estimated GFR [eGFR] < 60 mL/min/1.73 m^2^), proteinuria, osteoporosis and anemia. Serum chemistries, including complete metabolic panel and SUA levels, were routinely assessed before surgery as a part of the preoperative evaluation and during postoperative follow up; eGFR was calculated using the modification of diet in renal disease equation [16]. Serum uric acid at both institutions was quantified utilizing a Roche assay (Roche, Basel, Switzerland) and Cobas analyzer (Roche, Basel, Switzerland) that is based on an enzymatic colorimetric method developed by Town et al [31]. Demographics (age, sex, race, body mass index [BMI] and preoperative history of diabetes mellitus, hypertension, smoking and statins medications use), disease characteristics (American Joint Committee on Cancer, TNM classification) [32], and renal functional/metabolic outcomes (development of CKD defined as GFR < 60 mL/min per 1.73 m^2^, anemia defined as Hgb < 11.2 gm/dL [F], Hgb < 13.7 gm/dL [M], proteinuria defined as ≥1+ on urinalysis, osteoporosis defined as positive DEXA scan, and metabolic acidosis defined as HCO3^−^: <23 mEq/L) associated with decreased/stable SUA were recorded. 

### 4.3. Statistical Analysis

We defined ‘increased’ serum uric acid as an increase of greater than 10%, while all other values were defined as ‘decreased/stable’. Our classification was based on the meta-analysis of Ricos et al. who noted an 8.6% range of within-subject biological variability [33]. Furthermore, Yuki et al. found that a threshold of a 10% increase in SUA correlated with adverse oncological outcomes in RCC [20]. 

Analysis was carried out between two groups: patients with increased postoperative SUA vs. patients with decreased/stable postoperative SUA compared to pre-operative levels. Statistical analysis was conducted to identify factors that were significantly associated with decreased/stable SUA after radical nephrectomy (RN) or partial nephrectomy (PN). Variables were compared between the two groups (decreased/stable SUA vs. increased SUA) using Student’s *t*-test, ANOVA and Fisher’s exact/chi-squared tests for continuous and categorical variables, respectively. Kaplan-Meier analysis (KMA) was used to calculate overall survival (OS) and recurrence free survival (RFS) by comparing increased and decreased/stable SUA groups with log-rank test. Univariate logistic regression was performed to identify factors associated with decreased SUA and overall survival. All potential explanatory independent variables identified on univariate analysis were then further examined using multivariate stepwise logistic regression. Independent variables were included in the regression models if ≤0.10 on UVA to allow us to identify the adjusted variables that affected decreased SUA levels. The prognostic significance of variables for OS and RFS was analyzed by cox regression analysis. Pearson correlation coefficient analysis of clinical stage, increasing uric acid, and survival was also conducted. All *p* values were based on two-sided tests of significance, and *p* < 0.05 was considered to indicate statistical significance. Statistical analysis was performed using SAS version 9.1 (SAS Institute Inc., Cary, NC, USA).

## 5. Conclusions

This cohort study suggests the utility of SUA as marker for survival in RCC. Increasing SUA levels was associated with worsened outcomes in patients with RCC. Decreased SUA levels were associated with statins intake and lower stage disease as well as lack of CKD and anemia. Future studies are requisite to clarify the etiology of these interactions.

## Figures and Tables

**Table 1:** Demographics and disease characteristics in the decreased/stable uric acid and increased uric acid groups.

Variables	Uric Acid		*p*
	Decreased/Stable	Increased	
	(*n* = 675)	(*n* = 230)	
Age, years, mean ± SD	57 + 15.4	58 + 16	0.4734
Gender, *n* (%)			0.0393
Female	253 (37.5)	69 (30.0)	
Male	421 (62.5)	161 (70.0)	
Race, *n* (%)			0.8273
Caucasian	373 (55.3)	129 (56.1)	
Other	302 (44.7)	101 (43.9)	
Smoking History, *n* (%)	423 (62.7)	149 (64.8)	0.7366
BMI, kg/m^2^, mean ± SD	27.5 + 4.7	28.5 + 6.0	0.0201
History of DM, *n* (%)	148 (21.9)	62 (27.0)	0.1186
History of HTN, *n* (%)	412 (61.0)	150 (65.2)	0.2591
Statin Medications, *n* (%)	188 (27.9)	42 (18.3)	0.0039
ASA Class, *n* (%)			0.0662
2	264 (42.3)	78 (35.6)	
3	282 (45.1)	101 (46.1)	
4	79 (12.6)	40 (18.3)	
AJCC Clinical Stage, *n* (%)			<0.001
I	473 (70.1)	127 (55.2)	
II	128 (19.0)	46 (20.0)	
III	67 (9.9)	41 (17.8)	
IV	7 (1.0)	16 (7.0)	
Pathology, *n* (%)			0.0986
Clear Cell	510 (75.6)	186 (80.9)	
Other	165 (24.4)	44 (19.1)	
Surgery Type, *n* (%)			0.0002
Radical	432 (64.0)	178 (77.4)	
Partial	243 (36.0)	52 (22.6)	
Preop SUA	5.45 +2.13	5.59 + 1.243	0.345
Postop SUA	5.173 +1.21	6.42 +1.20	<0.001

BMI, Body mass index; DM, Diabetes mellitus; HTN, hypertension; ASA, American society of Anaesthesiologists’ physical status classification; AJCC, American Joint Committee on Cancer.

**Table 2:** Renal function and metabolic outcomes in the decreased/stable uric acid and increased uric acid groups.

Variable	Uric Acid		*p*
	Decreased/Stable	Increased	
	(*n* = 675)	(*n* = 230)	
Preop eGFR < 60	76 (11.3)	45 (19.6)	0.0014
Postop eGFR < 60	203 (30.1)	136 (59.1)	<0.0001
De Novo eGFR < 60	124 (18.4)	89 (38.7)	<0.0001
Preop Proteinuria	72 (10.7)	33 (14.4)	0.1322
Postop Proteinuria	140 (20.7)	71 (30.9)	0.0017
Preop Met Acidosis	22 (3.6)	12 (5.2)	0.1774
Postop Met Acidosis	79 (11.7)	48 (20.9)	0.0005
Preop Osteoporosis	55 (8.2)	27 (11.7)	0.2611
Postop Osteoporosis	110 (16.3)	66 (28.7)	<0.0001
Preop Anemia	106 (15.7)	55 (23.9)	0.0049
Postop Anemia	171 (25.3)	108 (47.0)	<0.0001

**Table 3:** Uni and multivariable regression analysis for factors associated with increased postoperative uric acid.

Variable	Univariate Analysis			Multivariate Analysis		
	OR	95% CI (Lower–Upper)	*p*	OR	95% CI (Lower–Upper)	*p*
Age (increasing)	1.004	0.994–1.013	0.473			
Sex, male (female ref.)	1.402	1.016–1.935	0.040	1.127	0.790–1.608	0.509
BMI (increasing)	1.039	1.009–1.069	0.009	1.049	1.012–1.088	0.009
ASA Score (increasing)	1.199	0.910–1.579	0.198			
Statin use (positive)	0.579	0.398–0.842	0.004	0.106	0.059–0.193	<0.001
Dyslipidemia (positive)	1.616	1.076–2.426	0.021	2.661	1.361–5.200	0.004
Stage II	1.338	0.906–1.976	0.143	1.123	0.720–1.750	0.610
Stage III	2.279	1.475–3.522	<0.001	1.887	1.131–3.148	0.015
Stage IV	8.513	3.428–21.139	<0.001	10.779	4.074–28.518	<0.001
RN (PN ref.)	1.925	1.361–2.723	<0.001	1.043	0.631–1.456	0.843
De novo CKD stage III	3.364	2.467–4.587	<0.001	5.952	3.954–8.961	<0.001
Preop SUA	1.035	0.963–1.112	0.353			

OR, Odds Ratio.

**Table 4:** Uni- and multivariate regression analysis of risk factors for all-cause mortality.

Variable	Univariate Analysis			Multivariate Analysis		
	OR	95% CI (Lower–Upper)	*p*	OR	95% CI (Lower–Upper)	*p*
Age (increasing)	1.008	0.999–1.017	0.081	0.989	0.970–1.009	0.267
Sex, male (female ref.)	1.459	1.081–1.969	0.014	1.352	0.844–2.166	0.210
BMI (increasing)	1.103	1.072–1.135	<0.001	1.085	1.030–1.142	0.002
ASA Score (increasing)	1.870	1.426–2.450	<0.001	1.578	1.098–2.268	0.014
Increased SUA (dec./stable SUA ref.)	10.068	7.154–14.170	<0.001	4.698	2.943–7.498	<0.001
Stage II	1.628	1.138–2.328	0.008	1.280	0.844–1.940	0.245
Stage III	2.412	1.585–3.669	<0.001	1.543	0.949–2.511	0.080
Stage IV	6.394	2.583–15.831	<0.001	7.702	2.876–20.629	<0.001
RN (PN ref.)	3.040	2.157–4.283	<0.001	1.620	1.084–2.421	0.019
de novo CKD stage III	7.618	5.571–10.418	<0.001	7.068	5.093–9.810	<0.001

**Table 5:** Cox proportional hazard analysis of prognostic factors for overall survival.

Variable	Univariate Analysis			Multivariate Analysis		
	HR	95% CI (Lower–Upper)	*p*	HR	95% CI (Lower–Upper)	*p*
Age (increasing)	1.011	1.004–1.019	0.003	1.017	1.005–1.028	0.004
Sex, male (female ref.)	1.364	1.060–1.756	0.016	1.051	0.803–1.376	0.718
BMI (increasing)	1.095	1.073–1.118	<0.001	1.099	1.070–1.129	<0.001
ASA Score (increasing)	1.731	1.465–2.046	<0.001	1.085	0.843–1.395	0.527
Increased SUA (dec./stable SUA ref.)	6.467	5.088–8.219	<0.001	4.708	3.647–6.078	<0.001
Stage II	1.541	1.152–2.061	0.004	1.336	0.981–1.820	0.066
Stage III	2.025	1.472–2.785	<0.001	1.537	1.095–2.157	0.013
Stage IV	7.119	4.219–12.014	<0.001	3.299	1.919–5.674	<0.001
RN (PN ref.)	2.172	1.600–2.948	<0.001	1.497	1.042–2.150	0.029
de novo CKD stage III	2.534	1.997–3.215	<0.001	1.684	1.297–2.186	<0.001

HR, Hazard Ratio.

**Table 6:** Cox proportional hazard analysis of prognostic factors for recurrence free survival.

Variable	Univariate Analysis			Multivariate Analysis		
	HR	95% CI (Lower–Upper)	*p*	HR	95% CI (Lower–Upper)	*p*
Age (increasing)	1.008	0.997–1.019	0.144			
Sex, male (female ref.)	1.545	1.069–2.233	0.021	0.983	0.656–1.473	0.933
BMI (increasing)	1.037	1.004–1.072	0.030	1.028	0.993–1.064	0.121
ASA Score (increasing)	1.398	1.101–1.776	0.006	1.239	0.954–1.609	<0.001
Increased SUA (dec./stable SUA ref.)	12.826	8.742-18.818	<0.001	9.782	6.479–14.77	<0.001
Stage II	2.725	1.791–4.147	<0.001	2.497	1.584–3.937	<0.001
Stage III	3.682	2.362–5.741	<0.001	3.195	1.992–5.127	<0.001
Stage IV	16.458	9.857–27.480	<0.001	6.911	3.953–12.082	<0.001
RN (PN ref.)	2.146	1.309–3.519	0.002	1.102	0.610–1.990	0.748
de novo CKD stage III	1.634	1.160–2.303	0.005	0.838	0.575–1.221	0.357

**Table 7:** Pearson correlation coefficient analysis of clinical stage, increased uric acid, and survival.

Variable	Clinical Stage	Increasing SUA	Survival (Months)
Clinical Stage	1		
Increased SUA	0.188 (*p* < 0.001)	1	
Survival (months)	−0.158 (*p* < 0.001)	−0.317 (*p* < 0.001)	1
